# Supplementary material for: Adaptive spatial-temporal information processing based on in-memory attention-inspired devices
Source: Nat Commun. 2025 Aug 12;16:7449. doi: 10.1038/s41467-025-62868-7 (PMC12343821; doi:10.1038/s41467-025-62868-7)
Supplement: Supplementary file 1 — Supplementary Information [file 41467_2025_62868_MOESM1_ESM.pdf]

Supplementary Information

Adaptive spatial-temporal processing based on in-memory attention-inspired devices

Jiong Pan[1,2,†], Fan Wu[1,2,3,†], Kangan Qian[4,5,†], Kun Jiang[4,5], Yanming Liu[1,2], Zeda Wang[1,2], Pengwen Guo[1,2], Jiaju Yin[1,2], Diange Yang[4,5,*], He Tian[1,2,*] , Yi Yang[1,2,*] and Tian-Ling Ren[1,2,*]

[1]School of Integrated Circuits, Tsinghua University, Beijing 100084, China.

[2]Beijing National Research Center for Information Science and Technology (BNRist), Tsinghua University, Beijing 100084, China.

[3]Shanghai Frontiers Science Research Base of Intelligent Optoelectronics and Perception, Institute of Optoelectronics, College of Future Information Technology, Fudan University, Shanghai 200433, China.

[4]School of Vehicle and Mobility, Tsinghua University, Beijing 100084, China

[5]State Key Laboratory of Intelligent Green Vehicle and Mobility, Tsinghua University, Beijing 100084, China

[†]These authors contributed equally: Jiong Pan, Fan Wu, Kangan Qian

*e-mails: RenTL@tsinghua.edu.cn; ydg@tsinghua.edu.cn; yiyang@tsinghua.edu.cn; tianhe88@tsinghua.edu.cn

Table of Contents

Supplementary Figure 1. MoS_2 channel transport behavior of 10 typical devices.

Supplementary Figure 2. Forward and backward scan curves of MoS_2 channel.

Supplementary Note 1. Filament state transfer tests.

Supplementary Figure 3. State transfer waveform by pulse signals.

Supplementary Figure 4. State transfer time of the attention-inspired device.

Supplementary Figure 5. Retention and endurance testing.

Supplementary Table 1. Comparisons of memristive devices for neuromorphic computing.

Supplementary Note 2. Discussions on device function stability.

Supplementary Figure 6. Energy band diagrams of the voltage configurations in attention distribution computing.

Supplementary Note 3. Shunt current analyses in computing mode.

Supplementary Figure 7. Shunt currents of the attention-inspired device varying with V_{IN}.

Supplementary Note 4. Analyses of electrostatic modulation stability.

Supplementary Figure 8. Transport behavior of the attention-inspired device.

Supplementary Figure 9. Circuit schematic of the 5×5 attention-inspired device array.

Supplementary Table 2. The output determination current I_{det} in different situations.

Supplementary Figure 10. Frame data of the 5×5 data stream input to the attention-inspired device array.

Supplementary Figure 11. Source data matrices of output current I_{out}.

Supplementary Figure 12. Statistical analyses of the attention-inspired device.

Supplementary Note 5. Implementation of the attention-enhanced edge intelligence.

Supplementary Figure 13. Modeling of the maximum source current I_{S0}.

Supplementary Table 3. Exponential fitting parameters.

Supplementary Figure 14. Flow chart of the attention-enhanced edge intelligence.

Supplementary Table 4. The spatial attention adjusted by CG voltage.

Supplementary Table 5. Situations in distinct scenes.

Supplementary Table 6. Spatial attention and CG voltage configurations in different scenes.

Supplementary Note 6. Spatial-temporal information recognition of attention-enhanced equipment.

Supplementary Figure 15. Workflow of integrating attention-enhanced equipment with VLMs for spatial-temporal information recognition in dynamic traffic scenes by utilizing a chain-of-thought process.

Supplementary Figure 16. Spatial and temporal information recognition analysis results.

Supplementary Note 7. Attention-inspired device performance analyses.

Supplementary References

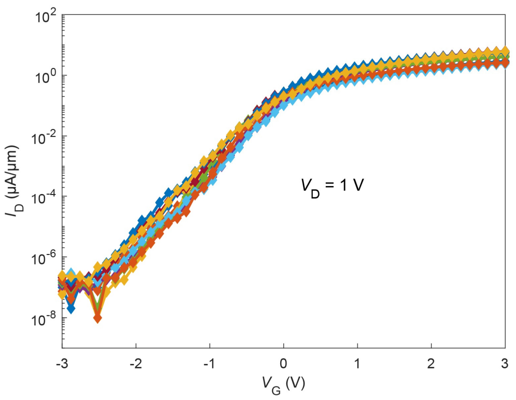

Supplementary Figure 1. MoS_2 channel transport behavior of 10 typical devices. V_D = 1 V. The channel length is 2 μm.

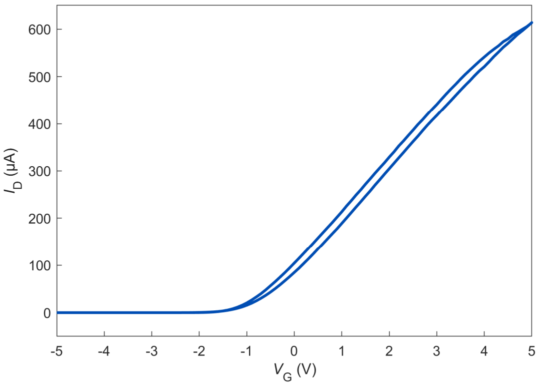

Supplementary Figure 2. Forward and backward scan curves of MoS_2 channel. V_D = 1 V. The channel width is 50 μm, and the channel length is 2 μm. The ratio (1.066) of the forward and backward scans is largely less than the state transfer current ratio (10^9).

Supplementary Note 1. Filament state transfer tests

Pulse data processing of the attention-inspired device in writing mode has been verified by experiments of the filament state transfer operated by pulse signals. V_D = 0.00 V, V_{CG} = 5.00 V. The waveform of state transfer is shown in Supplementary Fig. 3, illustrating stable and repeatable state transfer cycles. State transfer time is shown in Supplementary Fig. 4. The on/off states of I_{IN} before and after pulses indicate expected transfers between w\o-filament and w\-filament states. Filament state retention and endurance tests are conducted and shown in Supplementary Fig. 5. Performance specifications of this work and typical memristive device for neuromorphic computing are listed in Supplementary Table 1.

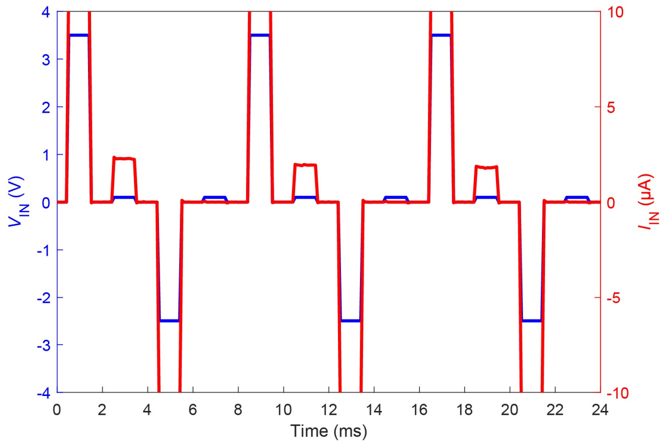

Supplementary Figure 3. State transfer waveform by pulse signals.

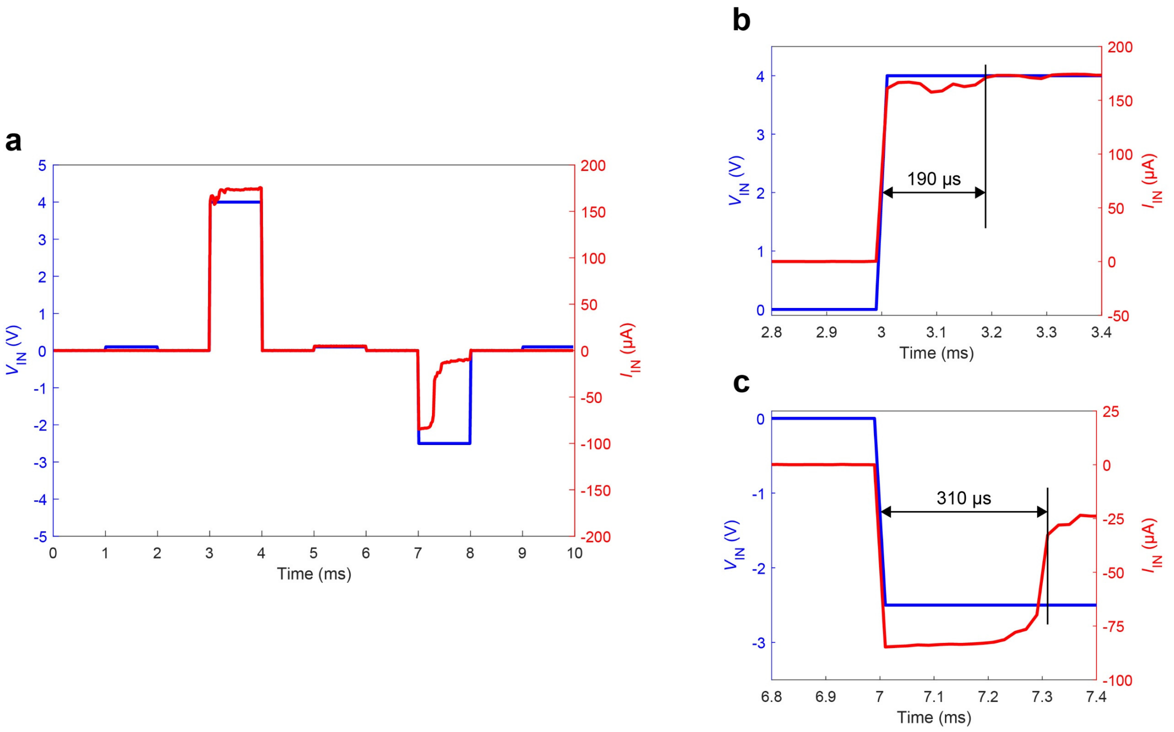

Supplementary Figure 4. State transfer time of the attention-inspired device. (a) A cycle of filament forming and rupture. (b) Enlarged waveform of filament forming. (c) Enlarged waveform of filament rupture.

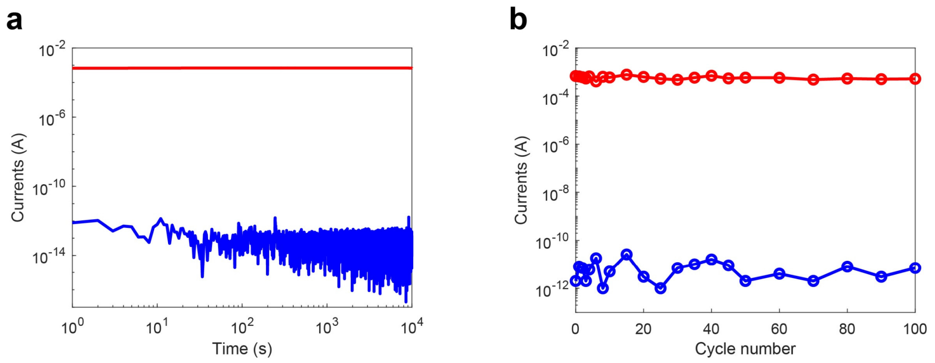

Supplementary Figure 5. (a) Retention and (b) endurance testing. The read voltage is 0.1 V.

Supplementary Table 1. Comparisons of memristive devices for neuromorphic computing.

References	Endurance	Retention	Switching time	On/Off ratio
Nature 2023[1]	10^6 cycles	10^2 ~ 10^4 s	232 μs	10^2
Nature Materials 2023[2]	10^4 cycles	10^2 s	\	10^{10}
Nature Communications 2019[3]	10^2 cycles	10^4 s	700 ns	10^3
Nature Communications 2022[4]	10^7 cycles	10^5 s	100 ns	10^2
Nature Communications 2023[5]	10^2 cycles	10^4 s	60 ns	10^2
Nature Communications 2024[6]	10^5 cycles	\	ms	2.4
This work	10^2 cycles	10^4 s	310 μs	10^9

Supplementary Note 2. Discussions on device function stability

As the increase of the switching cycles, defects are introduced to the dielectric film. When the defect density gradually becomes large, a conductive path would be formed through the dielectric, and the dielectric would be broken down, leading to the switching function degradation. Several strategies have been reported to mitigate the degradation and enlarge the endurance, including adding a stacking layer structure[7], widening the intrinsic threading

dislocation that acts as the preferential diffusion paths of Ag in the dielectric[8], reducing the quantity and movement of metal ions during switching via partial filament formation and variations[9], etc.

There are external factors influencing the device stability during fabrication processes or from the surroundings. Protons or moisture effects are one of the main sources influencing the dielectric film properties, since the filament-forming and rupture processes are sensitive to moisture. H_2O molecules are incorporated by absorption within the oxide with or without chemical interaction/dissociation, or defect-chemical reaction that introduces protons within the oxide[10]. Moisture has multiple effects on the dielectric film and device properities. The presence of H_2O molecules enables the filament formation, and affects the forming voltage[11-13]. Moisture also affects the switching behavior and set/reset kinetics[14-16]. It has been reported that moisture effects are relevant to matrix film properties including density and composition[17,18]. Furthermore, the retention performance is expected to be reduced with the increase of humidity[10,13,19]. To enhance the devices' stability, the effects of moisture should be controlled.

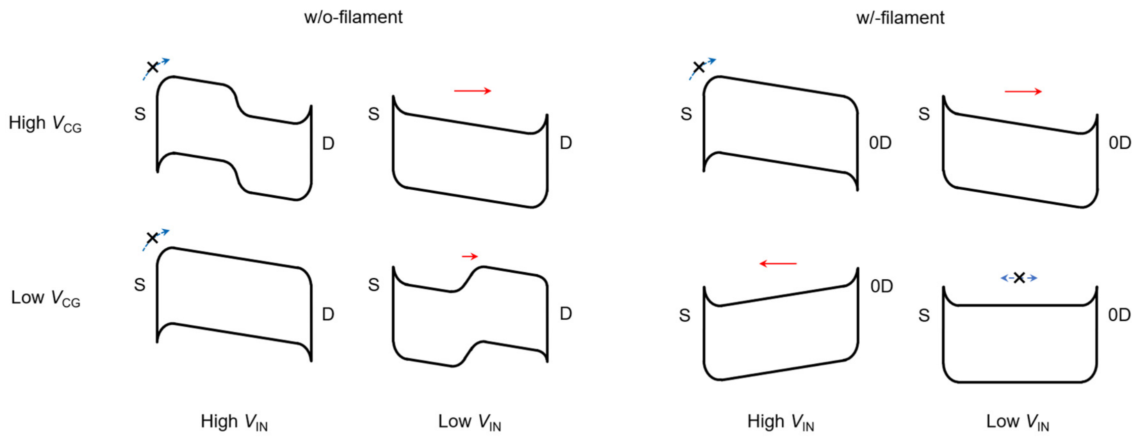

Supplementary Figure 6. Energy band diagrams of the voltage configurations in attention distribution computing. "S" denotes the source terminal, "D" denotes the drain terminal, and "0D" denotes the 0D interface.

Supplementary Note 3. Shunt current analyses in computing mode

I_{IN} at the Ag electrode is induced by 0D interface and flows through the filament as the shunt current of the channel. In attention distribution computing, shunt currents of 0D interface corresponding to Fig. 2f are shown in Supplementary Fig. 7a. Off-state shunt currents in w/o-

filament state and on-state shunt currents in w/-filament state are exhibited. Therefore, in w/o-filament state, 2D electrostatic modulation is not adjusted by shunt currents. In w/-filament state, I_{IN} and V_{IN} exhibit linear relation. V_{CG} controls the slopes of $I_{IN}-V_{IN}$ characteristics curves to adjust spatial and temporal attention, thereby modulating the transport behavior of I_S. In determination computing, shunt currents corresponding to Fig. 2i are shown in Supplementary Fig. 7b. Shunt current is off in w/o-filament state. When V_{IN} is positive, the source current is negatively high. In w/-filament state, when V_{IN} is negative, the induced positive shunt current offsets the drain current. When V_{IN} is positive, the negative shunt current generates the positive source current.

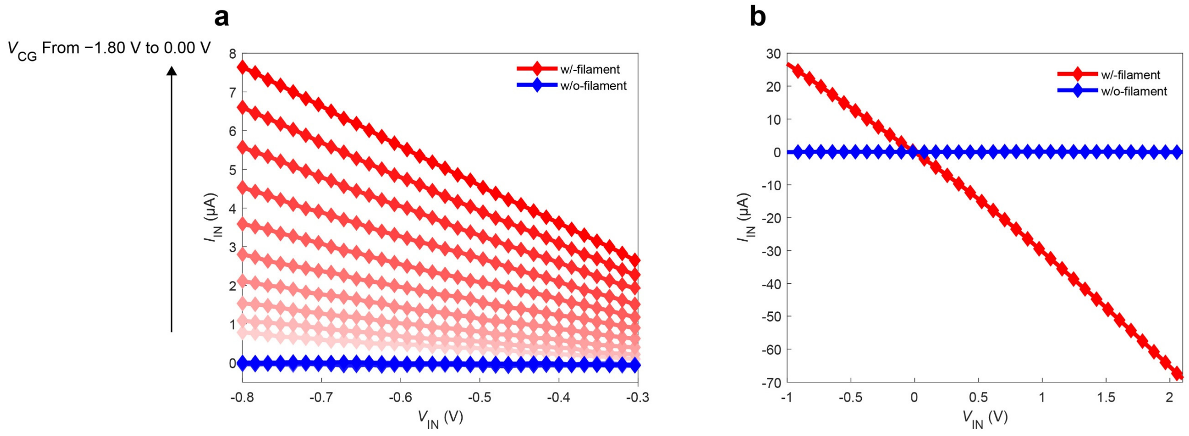

Supplementary Figure 7. Shunt currents of the attention-inspired device varying with V_{IN}. (a) $I_{IN}-V_{IN}$ characteristics of attention distribution computing corresponding to Fig. 2f. (b) $I_{IN}-V_{IN}$ characteristics of determination computing corresponding to Fig. 2i.

Supplementary Note 4. Analyses of electrostatic modulation stability

Electrostatic modulation stability of the Ag electrode to the 2D channel during transfer cycles between w/o- and w/-filament states ensures computing functionalities of the attention-inspired device. We characterized the I_S-V_{IN} transport behavior after multiple times of state transfers (Supplementary Fig. 8). The filament-forming scanning voltage is from 0.00 to 3.50 V, and the filament-rupture voltage is from 0.00 to −1.50 V. Transport curves were measured in w\o-filament state. The transport behavior is maintained after 100 times of state transfer cycles.

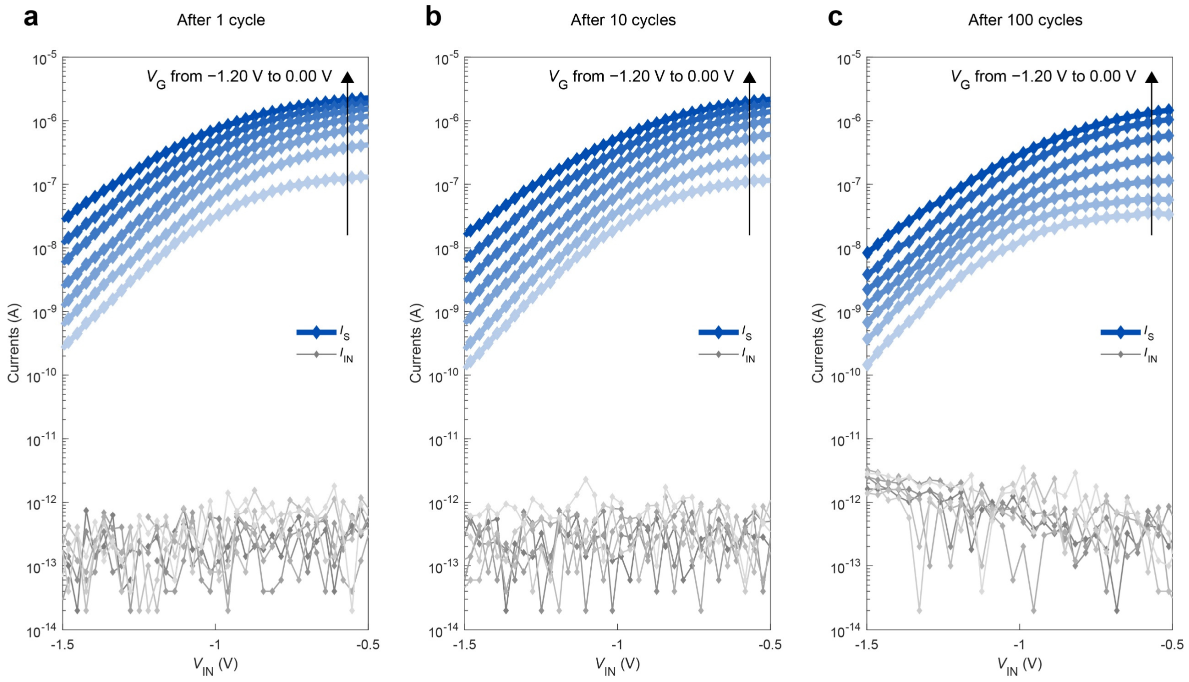

Supplementary Figure 8. Transport behavior of the attention-inspired device after (a) 1, (b) 10, and (c) 100 times of state transfer cycles. $V_D = 1.00$ V. $V_S = 0.00$ V. $V_{CG} = V_B = V_G$. V_{CG} and V_B are synchronically varied from −1.20 V to 0.00 V.

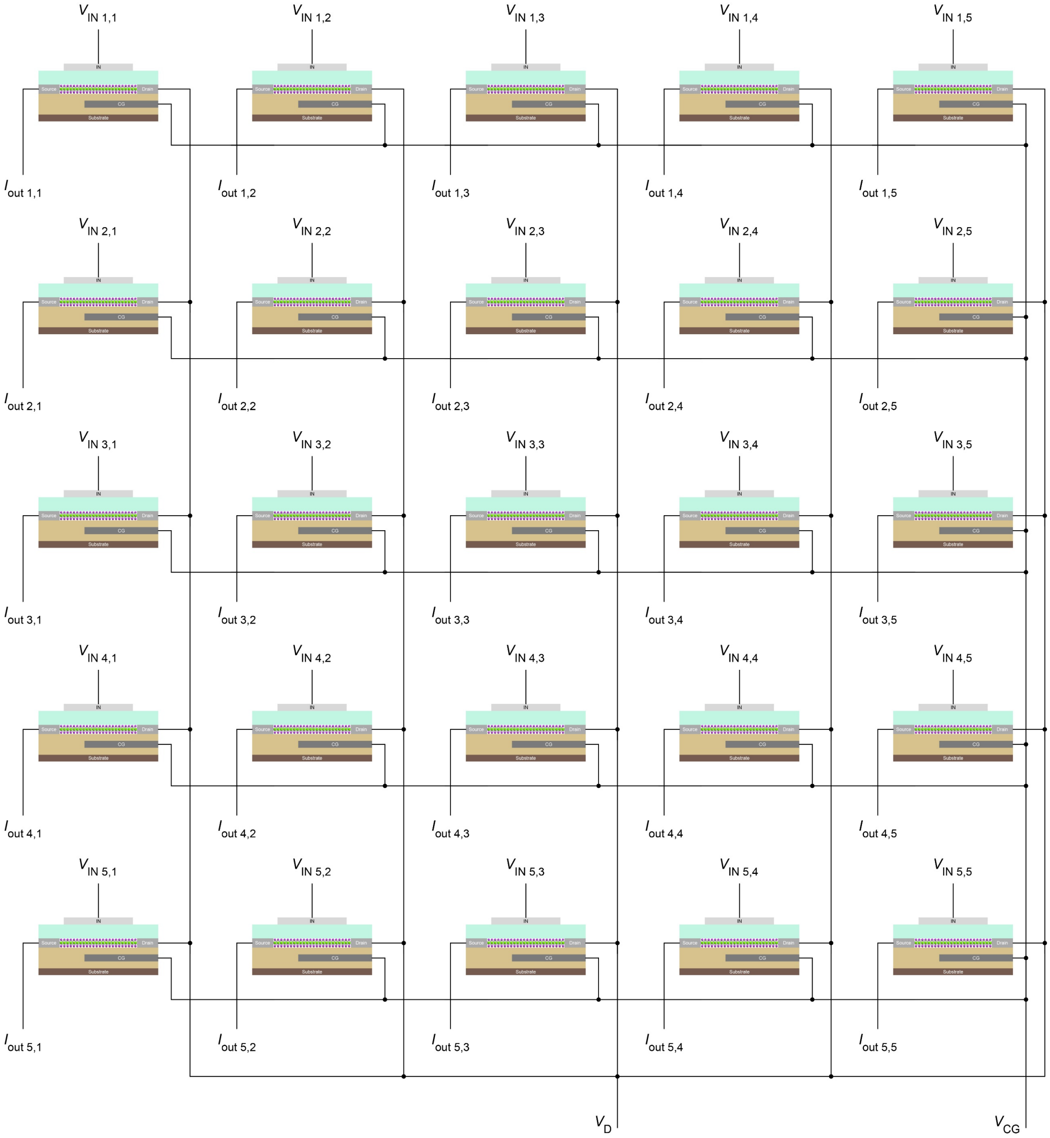

Supplementary Figure 9. Circuit schematic of the 5×5 attention-inspired device array. $V_{\text{IN}\ i,j}$ and $I_{\text{out}\ i,j}$ represent the input voltage and output current of the (i,j) pixel.

Supplementary Table 2. The output determination current I_{det} in different situations. I_{det} listed in the table are averaged values from Fig. 3b in 0.5 ms intervals for each situation value: [0.5, 1), [1.5, 2), [2.5, 3), and [3.5, 4) ms.

Situations		I_{det} (μA)
Spatial info. demand	Temporal info. demand	
T	T	−0.08
T	F	2.99
F	F	0.00
F	T	−2.81

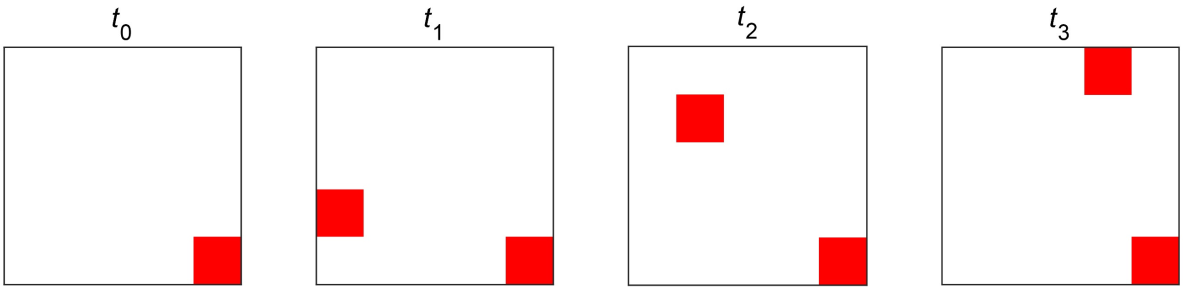

Supplementary Figure 10. Frame data of the 5×5 data stream input to the attention-inspired device array. White (logic 0) and red (logic 1) blocks represent input data values at each pixel. The data of t_0 defines the initial state of the attention-inspired device array. The interval of data processing is from t_1 to t_3.

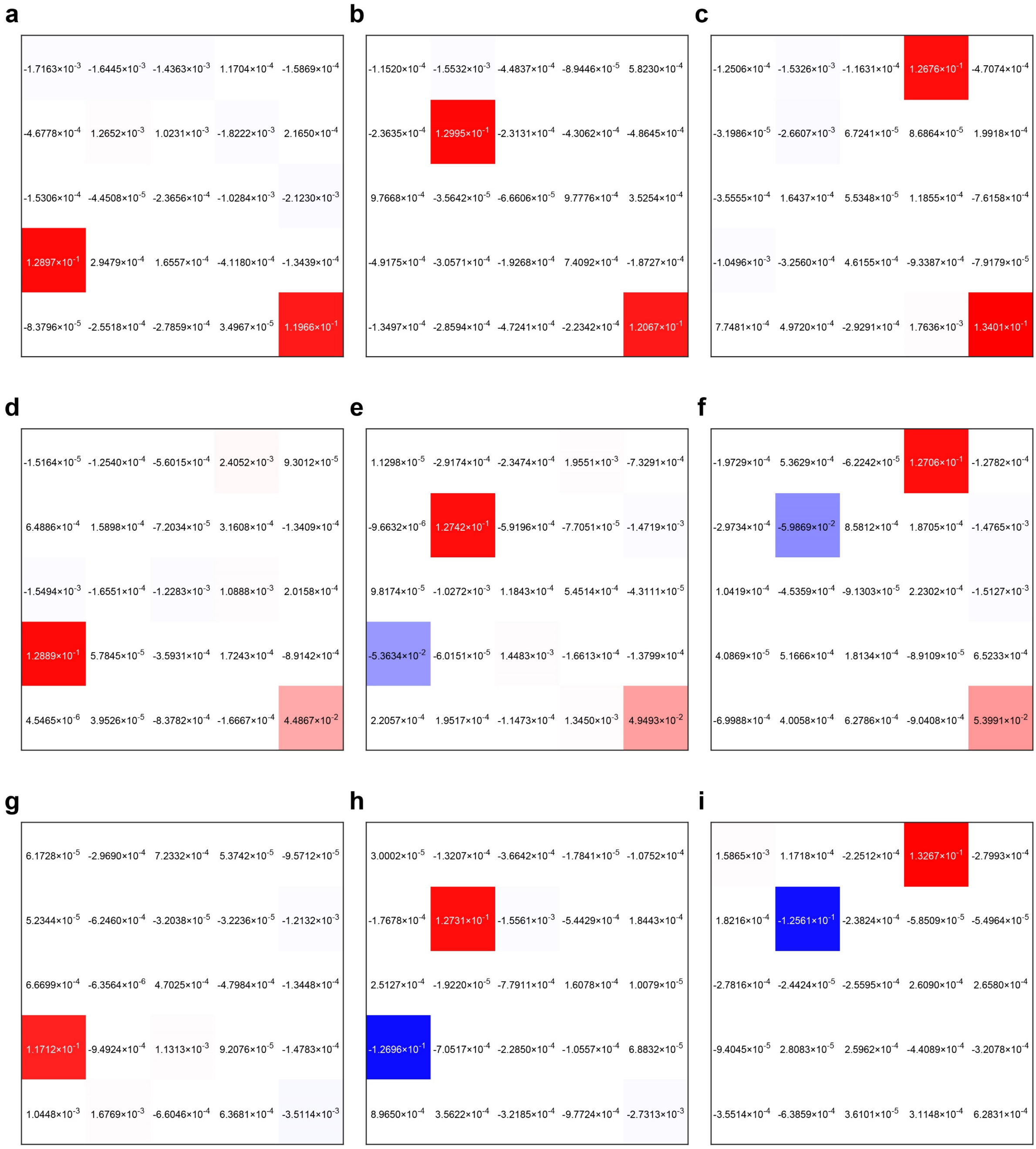

a

-1.7163×10^{-3}	-1.6445×10^{-3}	-1.4363×10^{-3}	1.1704×10^{-4}	-1.5869×10^{-4}
-4.6778×10^{-4}	1.2652×10^{-3}	1.0231×10^{-3}	-1.8222×10^{-3}	2.1650×10^{-4}
-1.5306×10^{-4}	-4.4508×10^{-5}	-2.3656×10^{-4}	-1.0284×10^{-3}	-2.1230×10^{-3}
1.2897×10^{-1}	2.9479×10^{-4}	1.6557×10^{-4}	-4.1180×10^{-4}	-1.3439×10^{-4}
-8.3796×10^{-5}	-2.5518×10^{-4}	-2.7859×10^{-4}	3.4967×10^{-5}	1.1966×10^{-1}

b

-1.1520×10^{-4}	-1.5532×10^{-3}	-4.4837×10^{-4}	-8.9446×10^{-5}	5.8230×10^{-4}
-2.3635×10^{-4}	1.2995×10^{-1}	-2.3131×10^{-4}	-4.3062×10^{-4}	-4.8645×10^{-4}
9.7668×10^{-4}	-3.5642×10^{-5}	-6.6606×10^{-5}	9.7776×10^{-4}	3.5254×10^{-4}
-4.9175×10^{-4}	-3.0571×10^{-4}	-1.9268×10^{-4}	7.4092×10^{-4}	-1.8727×10^{-4}
-1.3497×10^{-4}	-2.8594×10^{-4}	-4.7241×10^{-4}	-2.2342×10^{-4}	1.2067×10^{-1}

c

-1.2506×10^{-4}	-1.5326×10^{-3}	-1.1631×10^{-4}	1.2676×10^{-1}	-4.7074×10^{-4}
-3.1986×10^{-5}	-2.6607×10^{-3}	6.7241×10^{-5}	8.6864×10^{-5}	1.9918×10^{-4}
-3.5555×10^{-4}	1.6437×10^{-4}	5.5348×10^{-5}	1.1855×10^{-4}	-7.6158×10^{-4}
-1.0496×10^{-3}	-3.2560×10^{-4}	4.6155×10^{-4}	-9.3387×10^{-4}	-7.9179×10^{-5}
7.7481×10^{-4}	4.9720×10^{-4}	-2.9291×10^{-4}	1.7636×10^{-3}	1.3401×10^{-1}

d

-1.5164×10^{-5}	-1.2540×10^{-4}	-5.6015×10^{-4}	2.4052×10^{-3}	9.3012×10^{-5}
6.4886×10^{-4}	1.5898×10^{-4}	-7.2034×10^{-5}	3.1608×10^{-4}	-1.3409×10^{-4}
-1.5494×10^{-3}	-1.6551×10^{-4}	-1.2283×10^{-3}	1.0888×10^{-3}	2.0158×10^{-4}
1.2889×10^{-1}	5.7845×10^{-5}	-3.5931×10^{-4}	1.7243×10^{-4}	-8.9142×10^{-4}
4.5465×10^{-6}	3.9526×10^{-5}	-8.3782×10^{-4}	-1.6667×10^{-4}	4.4867×10^{-2}

e

1.1298×10^{-5}	-2.9174×10^{-4}	-2.3474×10^{-4}	1.9551×10^{-3}	-7.3291×10^{-4}
-9.6632×10^{-6}	1.2742×10^{-1}	-5.9196×10^{-4}	-7.7051×10^{-5}	-1.4719×10^{-3}
9.8174×10^{-5}	-1.0272×10^{-3}	1.1843×10^{-4}	5.4514×10^{-4}	-4.3111×10^{-5}
-5.3634×10^{-2}	-6.0151×10^{-5}	1.4483×10^{-3}	-1.6613×10^{-4}	-1.3799×10^{-4}
2.2057×10^{-4}	1.9517×10^{-4}	-1.1473×10^{-4}	1.3450×10^{-3}	4.9493×10^{-2}

f

-1.9729×10^{-4}	5.3629×10^{-4}	-6.2242×10^{-5}	1.2706×10^{-1}	-1.2782×10^{-4}
-2.9734×10^{-4}	-5.9869×10^{-2}	8.5812×10^{-4}	1.8705×10^{-4}	-1.4765×10^{-3}
1.0419×10^{-4}	-4.5359×10^{-4}	-9.1303×10^{-5}	2.2302×10^{-4}	-1.5127×10^{-3}
4.0869×10^{-5}	5.1666×10^{-4}	1.8134×10^{-4}	-8.9109×10^{-5}	6.5233×10^{-4}
-6.9988×10^{-4}	4.0058×10^{-4}	6.2786×10^{-4}	-9.0408×10^{-4}	5.3991×10^{-2}

g

6.1728×10^{-5}	-2.9690×10^{-4}	7.2332×10^{-4}	5.3742×10^{-5}	-9.5712×10^{-5}
5.2344×10^{-5}	-6.2460×10^{-4}	-3.2038×10^{-5}	-3.2236×10^{-5}	-1.2132×10^{-3}
6.6699×10^{-4}	-6.3564×10^{-6}	4.7025×10^{-4}	-4.7984×10^{-4}	-1.3448×10^{-4}
1.1712×10^{-1}	-9.4924×10^{-4}	1.1313×10^{-3}	9.2076×10^{-5}	-1.4783×10^{-4}
1.0448×10^{-3}	1.6769×10^{-3}	-6.6046×10^{-4}	6.3681×10^{-4}	-3.5114×10^{-3}

h

3.0002×10^{-5}	-1.3207×10^{-4}	-3.6642×10^{-4}	-1.7841×10^{-5}	-1.0752×10^{-4}
-1.7678×10^{-4}	1.2731×10^{-1}	-1.5561×10^{-3}	-5.4429×10^{-4}	1.8443×10^{-4}
2.5127×10^{-4}	-1.9220×10^{-5}	-7.7911×10^{-4}	1.6078×10^{-4}	1.0079×10^{-5}
-1.2696×10^{-1}	-7.0517×10^{-4}	-2.2850×10^{-4}	-1.0557×10^{-4}	6.8832×10^{-5}
8.9650×10^{-4}	3.5622×10^{-4}	-3.2185×10^{-4}	-9.7724×10^{-4}	-2.7313×10^{-3}

i

1.5865×10^{-3}	1.1718×10^{-4}	-2.2512×10^{-4}	1.3267×10^{-1}	-2.7993×10^{-4}
1.8216×10^{-4}	-1.2561×10^{-1}	-2.3824×10^{-4}	-5.8509×10^{-5}	-5.4964×10^{-5}
-2.7816×10^{-4}	-2.4424×10^{-5}	-2.5595×10^{-4}	2.6090×10^{-4}	2.6580×10^{-4}
-9.4045×10^{-5}	2.8083×10^{-5}	2.5962×10^{-4}	-4.4089×10^{-4}	-3.2078×10^{-4}
-3.5514×10^{-4}	-6.3859×10^{-4}	3.6101×10^{-5}	3.1148×10^{-4}	6.2831×10^{-4}

Supplementary Figure 11. Source data matrices of output current I_{out}. (a–i) correspond to Fig. 3e–m respectively. The unit of I_{out} is μA.

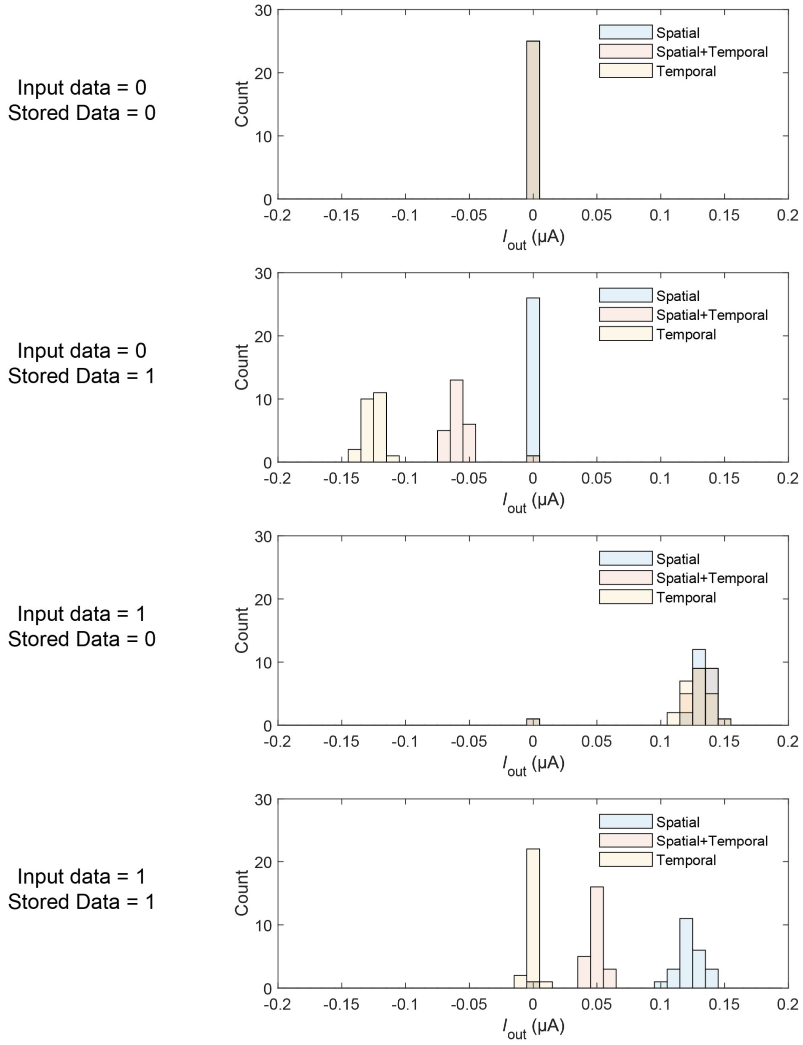

Supplementary Figure 12. Statistical analyses of the attention-inspired device. The output currents of 25 devices are measured with different input and stored data, and different attention configurations (spatial, spatial+temporal, and temporal). Among the 25 devices, 24 are functioning properly.

Supplementary Note 5. Implementation of the attention-enhanced edge intelligence

The model of V_{CG}-controlled attention adjustment is established by experimental data of the attention-inspired device. We conduct exponential fitting to obtain an empirical model of spatial and temporal attentions varied with V_{CG}. The model function is $y=a+be^{[c(x+d)]}$, where x is the value of V_{CG}, and y is the spatial or temporal attention. Then an empirical model of the maximum source current I_{S0} is built, as shown in Supplementary Fig. 13. The parameter values are listed in Supplementary Table 3. Mappings between V_{CG}, V_{IN}, and I_S are established by the

models and Equation (1) and (2). Given a value of the required attention, the value of V_{CG} is obtained (Supplementary Table 4), and then I_S is calculated by the established model.

The flow chart of the attention-enhanced edge intelligence implementation is shown in Supplementary Fig. 14. Various situations are considered in different scenes as the inputs of determination computing (Supplementary Table 5). "T" and "F" mean that the situation is true or false respectively. An $m \times n$ array is built to perform n-bit perception from the input situations. $m = 8$ is the number of situations. n is set to be 4 in this demonstration. The output determination currents $I_{\text{det1}} - I_{\text{det}n}$ are obtained by analog computing:

$$I_{\text{det}\,j} = I_{\text{e}} \cdot \sum_{i=1}^{m} (w_{i,j} \cdot x_i) \ , \qquad j = 1,\ 2,\ ...,\ n \tag{1}$$

where $x_i \in \{0, 1\}$ is the logic of the situation i. $i = 1, 2, \ldots, m$. The range of weight for each device cell is $w_{i,j} \in \{-1, 1\}$. $I_{\text{det1}} - I_{\text{det}n}$ are input to the post-processing module to obtain the determination output. Circuit designs of post-processing modules for analog computing include circuits composed of transimpedance amplifier (TIA), analog-to-digital converter (ADC), digital shifter, and adder[20], or circuits composed of TIA, analog summation node, and ADC[21]. Accumulation of determination currents in bit $1-n$ is conducted in the post-processing module to obtain the optimized value of attention in the given situations:

$$\alpha_{\text{spatial}} = \frac{\sum_{j=1}^{n} 2^j I_{\text{det}\,j}}{I_{\text{det}\,0}} \tag{2}$$

where I_{det0} is the normalization constant. α_{spatial} is clamped to the range [0%, 100%]. The values of spatial attention obtained in different scenes are listed in Supplementary Table 6.

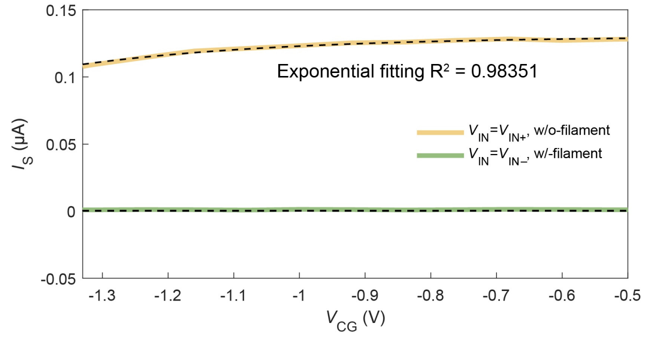

Supplementary Figure 13. Modeling of the maximum source current I_{S0}.

Supplementary Table 3. Exponential fitting parameters.

	Parameters			
	a	*b*	*c*	*d*
I_{S0}	0.130278	−0.0141960	−3.1706	1.20822
$\alpha_{spatial}$	1.06207	−0.85689	−3.3540	1.26730
$\alpha_{temporal}$	−0.0036477	1.84917	−5.2985	1.43504

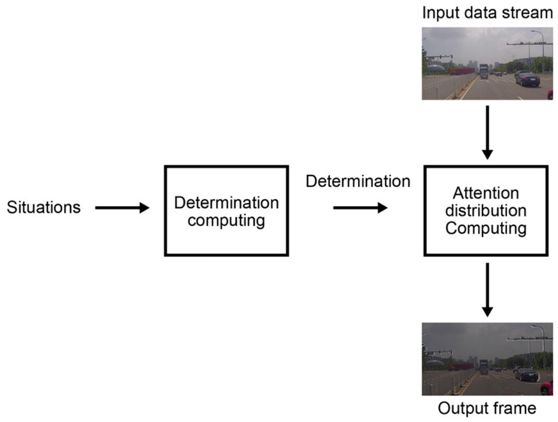

Supplementary Figure 14. Flow chart of the attention-enhanced edge intelligence.

Supplementary Table 4. The spatial attention adjusted by CG voltage.

V_{CG} (V)	−1.33	−1.27	−1.19	−1.08	−0.91	−0.48
$\alpha_{spatial}$	0%	20%	40%	60%	80%	100%

Supplementary Table 5. Situations in distinct scenes.

No.	Situations	Scene1<br>Wide street	Scene2<br>Congested street	Scene3<br>Crossroad
1	High speed	T	F	F
2	Heavy traffic	F	T	F
3	Bumpy road	F	F	F
4	Location recognition	F	F	T
5	Moving vehicle detection	T	T	F
6	Obstacle detection	F	T	F
7	Traffic light detection	F	F	T
8	Long-term parking	F	F	F

Supplementary Table 6. Spatial attention and CG voltage configurations in different scenes.

	Scene1<br>Wide street	Scene2<br>Congested street	Scene3<br>Crossroad
$\alpha_{spatial}$	0.5%	47.5%	90.0%
V_{CG} (V)	−1.32	−1.15	−0.77

Supplementary Note 6. Spatial-temporal information recognition of attention-enhanced equipment

Attention-enhanced equipment was connected to Vision-Language Models (VLMs) for spatial-temporal information recognition. A tool library was introduced that leverages neural modules to dynamically gather text-based environmental information. The cornerstone components of the tool library are two neural modules: a 2D detector[22] and a visual prompt generator. The 2D detector processes image data to generate bounding boxes around detected objects, while the visual prompt generator overlays these bounding boxes onto the images, providing clear visual cues.

A chain-of-thought[23] process was applied to analyze spatial and temporal information in dynamic traffic scenes (Supplementary Fig. 15). Initially, a proven 2D detector was employed to identify the location and category of objects within the image. Next, visualization code was designed to overlay these bounding boxes on the image using distinct colors based on object categories, serving as visual prompts to simplify caption generation in complex scenes. These prompts were then integrated into VLMs to enhance scene understanding and reasoning. This method breaks down the analysis of spatial and temporal information into simpler, more manageable steps, thereby improving interpretability and controllability. We used Vicuna-7B v1.5[24] as the large language model (LLM), with visual encoders initialized from OpenCLIP-L/14[25]. The text tokenizer was sourced from LLaMA[26], with approximately 32,000 classes. The unified visual representation was then passed through a shared projection layer before being fed into the LLM.

Qualitative results of the spatial and temporal information recognition analysis are illustrated in Supplementary Fig. 16. Our method accurately recognizes and captures both temporal information—such as a black SUV crossing the intersection—and spatial information like traffic lights. Moreover, our approach effectively comprehends the traffic rules within the

scene based on the complete spatial and temporal information extracted from the attention-enhanced equipment.

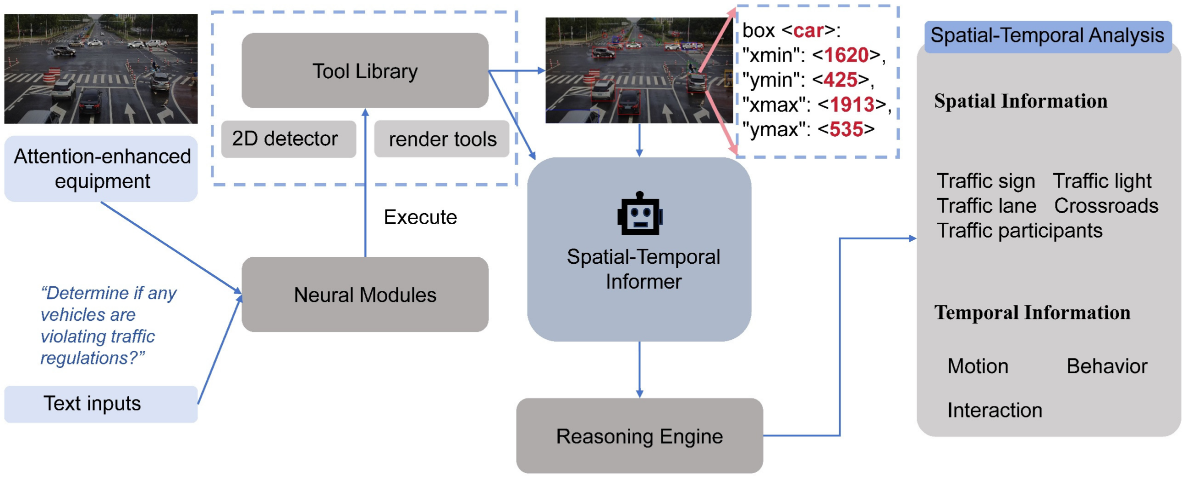

Supplementary Figure 15. Workflow of integrating attention-enhanced equipment with VLMs for spatial-temporal information recognition in dynamic traffic scenes by utilizing a chain-of-thought process.

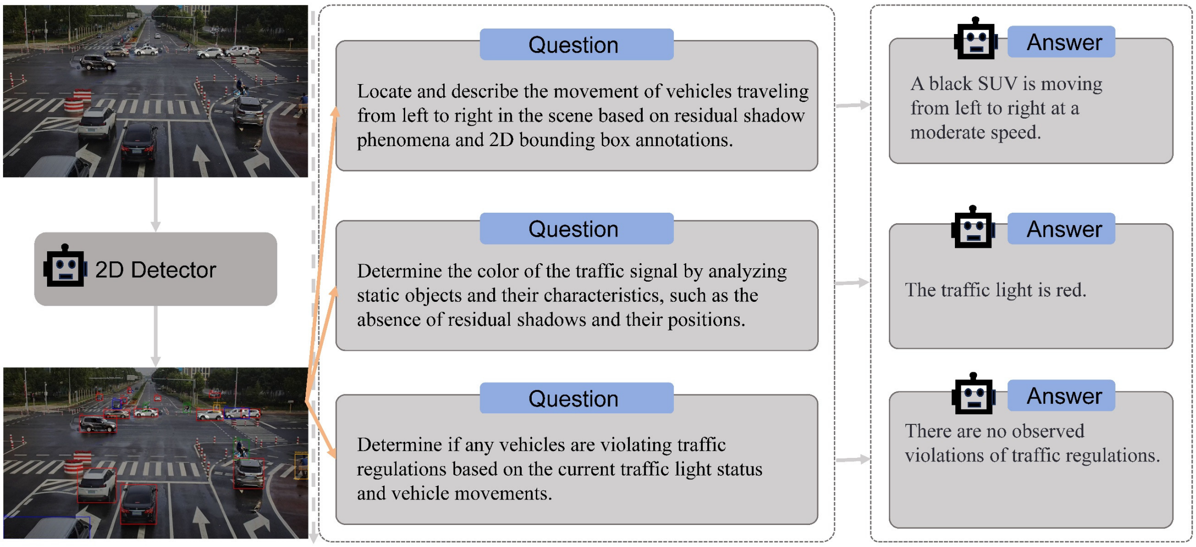

Supplementary Figure 16. Spatial and temporal information recognition analysis results.

Supplementary Note 7. Attention-inspired device performance analyses

To analyze the performance of the attention-inspired device, simulation tests of the adaptive spatial-temporal information processing circuit based on the attention-inspired device have been conducted to evaluate the latency and energy cost. A Verilog-A model of the attention-inspired device has been established. The modeling parameters are extracted by the experimental data of transfer and output curves. Then a schematic model of the device is encapsulated, and is referenced by the circuit-level simulation project. Pulse signals are input to the device to simulate the input data stream, and attention is adjusted by the CG voltage level. Propagation delay and averaged currents are measured. The value of latency equals the propagation delay, and the energy cost E is given by:

$$E = (|V_{\mathrm{IN}}\overline{I_{\mathrm{IN}}}| + |V_{\mathrm{S}}\overline{I_{\mathrm{S}}}| + |V_{\mathrm{D}}\overline{I_{\mathrm{D}}}|)\cdot t \tag{3}$$

where t is the latency, and $\overline{I_{\mathrm{IN}}}$, $\overline{I_{\mathrm{S}}}$, $\overline{I_{\mathrm{D}}}$ are averaged current at the IN, source, and drain terminals respectively. A comparison of the attention-inspired-device-based architecture to a standard CMOS circuit has been conducted. The CMOS circuit implementing the equivalent adaptive spatial-temporal information processing functionality needs to realize the following operation:

$$D_{\mathrm{out}}(t) = \alpha_{\mathrm{spatial}}\cdot D_{\mathrm{in}}(t) + \alpha_{\mathrm{temporal}}\cdot[D_{\mathrm{in}}(t) - D_{\mathrm{in}}(t-1)] \tag{4}$$

where $D_{\mathrm{in}}(t)$ and $D_{\mathrm{out}}(t)$ are input and output data respectively. Multiple digital arithmetic units are needed to implement the operation. For example, to implement 4-bit spatial and temporal attention adjustment, 5 NAND gates, 5 AND gates, and 5 full adders are required, even not considering the peripheral memory units storing the previous input data. A schematic model of the CMOS circuit is established. Latency and energy are measured by pulse signal tests. The latency and energy measurement results of the proposed architecture and the CMOS circuit at each attention value are shown in Fig. 4e−f. Since the attention-inspired device integrates multidimensional information processing into the device level, a large amount of device numbers is saved for spatial-temporal information processing. 4 transistors, 6 transistors, and 28 transistors are required for NAND gates, AND gates, full adders respectively[27]. Therefore, area of the CMOS circuit is:

$$A_{\mathrm{CMOS}} = 5\cdot A_{\mathrm{NAND}} + 5\cdot A_{\mathrm{AND}} + 5\cdot A_{\mathrm{adder}} = 190A_0 \tag{5}$$

where A_0 is the transistor area. The attention-inspired device has the same area as the transistor. Since the operation of each unit is performed by a single attention-inspired device, the area is reduced by 190 compared to the CMOS circuit.

Supplementary References

1. Zhu, K. et al. Hybrid 2D-CMOS microchips for memristive applications. *Nature* **618**, 57-62 (2023). https://doi.org/10.1038/s41586-023-05973-1
2. Kang, J.-H. et al. Monolithic 3D integration of 2D materials-based electronics towards ultimate edge computing solutions. *Nat. Mater.* **22**, 1470-1477 (2023). https://doi.org/10.1038/s41563-023-01704-z
3. Sivan, M. et al. All WSe_2 1T1R resistive RAM cell for future monolithic 3D embedded memory integration. *Nat. Commun.* **10**, 5201 (2019). https://doi.org/10.1038/s41467-019-13176-4
4. Tang, B. et al. Wafer-scale solution-processed 2D material analog resistive memory array for memory-based computing. *Nat. Commun.* **13**, 3037 (2022). https://doi.org/10.1038/s41467-022-30519-w
5. Xie, M. et al. Monolithic 3D integration of 2D transistors and vertical RRAMs in 1T-4R structure for high-density memory. *Nat. Commun.* **14**, 5952 (2023). https://doi.org/10.1038/s41467-023-41736-2
6. Park, J. et al. Multi-level, forming and filament free, bulk switching trilayer RRAM for neuromorphic computing at the edge. *Nat. Commun.* **15** (2024). https://doi.org/10.1038/s41467-024-46682-1
7. Yan, X. et al. Robust Ag/ZrO_2/WS_2/Pt Memristor for Neuromorphic Computing. *ACS Appl. Mater. Interfaces* **11**, 48029-48038 (2019). https://doi.org/10.1021/acsami.9b17160
8. Choi, S. et al. SiGe epitaxial memory for neuromorphic computing with reproducible high performance based on engineered dislocations. *Nat. Mater.* **17**, 335-340 (2018). https://doi.org/10.1038/s41563-017-0001-5
9. Kim, J., Kwon, O., Seo, J. & Hwang, H. Vertical-Switching Conductive Bridge Random Access Memory with Adjustable Tunnel Gap and Improved Switching Uniformity Using 2D Electron Gas. *Adv. Electron. Mater.* (2024). https://doi.org/10.1002/aelm.202400650
10. Valov, I. & Tsuruoka, T. Effects of moisture and redox reactions in VCM and ECM resistive switching memories. *J. Phys. D: Appl. Phys.* **51** (2018). https://doi.org/10.1088/1361-6463/aad581
11. Tappertzhofen, S., Hempel, M., Valov, I. & Waser, R. Proton mobility in SiO_2 thin films and impact of hydrogen and humidity on the resistive switching effect. *Mater. Res. Soc. Symp. Proc.* **1330** (2011). https://doi.org/https://doi.org/10.1557/opl.2011.1198

12. Tappertzhofen, S. et al. Generic relevance of counter charges for cation-based nanoscale resistive switching memories. *ACS Nano* **7**, 6396-6402 (2013). https://doi.org/10.1021/nn4026614

13. Tsuruoka, T., Hasegawa, T., Terabe, K. & Aono, M. Operating mechanism and resistive switching characteristics of two- and three-terminal atomic switches using a thin metal oxide layer. *J. Electroceramics* **39**, 143-156 (2017). https://doi.org/10.1007/s10832-016-0063-9

14. Tsuruoka, T. et al. Effects of Moisture on the Switching Characteristics of Oxide-Based, Gapless-Type Atomic Switches. *Adv. Funct. Mater.* **22**, 70-77 (2011). https://doi.org/10.1002/adfm.201101846

15. Chang, C.-F. et al. Direct Observation of Dual-Filament Switching Behaviors in Ta_2O_5-Based Memristors. *Small* **13**, 1603116 (2017). https://doi.org/https://doi.org/10.1002/smll.201603116

16. Lubben, M. et al. SET kinetics of electrochemical metallization cells: influence of counter-electrodes in SiO_2/Ag based systems. *Nanotechnology* **28**, 135205 (2017). https://doi.org/10.1088/1361-6528/aa5e59

17. Tsuruoka, T. et al. Redox Reactions at Cu,Ag/Ta_2O_5 Interfaces and the Effects of Ta_2O_5 Film Density on the Forming Process in Atomic Switch Structures. *Adv. Funct. Mater.* **25**, 6374-6381 (2015). https://doi.org/https://doi.org/10.1002/adfm.201500853

18. Mannequin, C., Tsuruoka, T., Hasegawa, T. & Aono, M. Identification and roles of nonstoichiometric oxygen in amorphous Ta_2O_5 thin films deposited by electron beam and sputtering processes. *Appl. Surf. Sci.* **385**, 426-435 (2016). https://doi.org/10.1016/j.apsusc.2016.04.099

19. Mannequin, C., Tsuruoka, T., Hasegawa, T. & Aono, M. Composition of thin Ta_2O_5 films deposited by different methods and the effect of humidity on their resistive switching behavior. *Jpn. J. Appl. Phys.* **55** (2016). https://doi.org/10.7567/jjap.55.06gg08

20. Yao, P. et al. Fully hardware-implemented memristor convolutional neural network. *Nature* **577**, 641-646 (2020). https://doi.org/10.1038/s41586-020-1942-4

21. Song, W. et al. Programming memristor arrays with arbitrarily high precision for analog computing. *Science* **383**, 903-910 (2024). https://doi.org/doi:10.1126/science.adi9405

22. Ren, S., He, K., Girshick, R. & Sun, J. Faster R-CNN: Towards Real-Time Object Detection with Region Proposal Networks. *IEEE Trans. Pattern Anal. Mach. Intell.* **39**, 1137-1149 (2017). https://doi.org/10.1109/TPAMI.2016.2577031

23. Wei, J. et al. Chain-of-thought prompting elicits reasoning in large language models. In *Proc. Proceedings of the 36th International Conference on Neural Information Processing Systems* Article 1800 (Curran Associates Inc., 2024).
24. Zheng, L. et al. Judging LLM-as-a-judge with MT-bench and Chatbot Arena. In *Proc. Proceedings of the 37th International Conference on Neural Information Processing Systems* Article 2020 (Curran Associates Inc., 2024).
25. Li, X., Wang, Z. & Xie, C. An inverse scaling law for CLIP training. In *Proc. Proceedings of the 37th International Conference on Neural Information Processing Systems* Article 2132 (Curran Associates Inc., 2024).
26. Touvron, H. et al. LLaMA: Open and Efficient Foundation Language Models. Preprint at https://arxiv.org/abs/2302.13971 (2023).
27. Rabaey, J. M., Chandrakasan, A. & Nikolić, B. *Digital integrated circuits: a design perspective* Ed. 2 (Pearson, 2002).
